# Advances in Tapered Optical Fiber Sensor Structures: From Conventional to Novel and Emerging

**DOI:** 10.3390/bios13060644

**Published:** 2023-06-12

**Authors:** Wen Zhang, Xianzheng Lang, Xuecheng Liu, Guoru Li, Ragini Singh, Bingyuan Zhang, Santosh Kumar

**Affiliations:** 1 Shandong Key Laboratory of Optical Communication Science and Technology, School of Physics Science and Information Technology, Liaocheng University, Liaocheng 252059, China; 2110110405@stu.lcu.edu.cn (W.Z.); grli@lcu.edu.cn (G.L.); 2 College of Agronomy, Liaocheng University, Liaocheng 252059, China; singh@lcu.edu.cn

**Keywords:** fiber-optic sensor, tapered optical fiber structure, physical sensors, chemical sensors, gas sensors, novel tapered optical fiber, humanoid tapered fiber structure

## Abstract

Optical fiber sensors based on tapered optical fiber (TOF) structure have attracted a considerable amount of attention from researchers due to the advantages of simple fabrication, high stability, and diverse structures, and have great potential for applications in many fields such as physics, chemistry, and biology. Compared with conventional optical fibers, TOF with their unique structural characteristics significantly improves the sensitivity and response speed of fiber-optic sensors and broadens the application range. This review presents an overview of the latest research status and characteristics of fiber-optic sensors and TOF sensors. Then, the working principle of TOF sensors, fabrication schemes of TOF structures, novel TOF structures in recent years, and the growing emerging application areas are described. Finally, the development trends and challenges of TOF sensors are prospected. The objective of this review is to convey novel perspectives and strategies for the performance optimization and design of TOF sensors based on fiber-optic sensing technologies.

## 1. Introduction

In the 1960s, a waveguide made of quartz was used for the first time to transmit optical signals, which became known as optical fibers. Corning has developed low-loss optical fibers that can transmit optical signals over long distances. This led to a period of rapid development of fiber-optic communication. Subsequently, fiber-optic sensing came into being. Compared with traditional electrical sensors, fiber-optic sensing uses optical signals as the modulation and transmission carrier, which allows it to have many unique advantages [1,2,3,4], such as strong resistance to electromagnetic interference in the transmission process, thus allowing it to play a very significant role in the power system [5,6], and strong corrosion resistance, which can be measured for highly corrosive analytes [7], as it has a compact structure that can be fabricated according to the needs of the size of very small fiber optic sensors [8]. Simultaneously, several studies have highlighted the advantages of simple fiber optic materials, cost-effectiveness, and broad scale utilization [9,10]. With their own inimitable characteristics, fiber-optic sensors have broad application prospects in a variety of sectors, including environmental monitoring, civil engineering, biomedicine, industrial production, aerospace, energy development, and food safety [11,12,13,14,15,16,17]. 

Optical fibers have excellent resistance to interference from the external environment, which enables them to be highly reliable and stable, and to a certain extent expands their application fields. In order to achieve the sensing function and increase the sensitivity, special processing procedures are frequently used to modify the geometry of the fiber in order to disrupt the original total reflection transmission mode. Processing methods include taper pulling, core-offset splicing, laser etching, and side grinding and polishing [8,18,19,20,21,22,23].

Through advanced optical fiber processing equipment and fabrication techniques, more and more special fiber structures are coming onto the stage of fiber sensing, such as tapered optical fiber (TOF) structure [17], D-shaped structure [24], U-shaped structure [25], S-shaped structure [26], fiber grating structure [27], heterocore structure [28], core-offset structure [29,30], and microsphere structure [31]. These special fiber structures can effectively excite the enhanced evanescent field and expose it to and interact with the surrounding medium, thus promoting the interaction between light and sensing materials and achieving higher sensing sensitivity. Among them, the TOF structure has a smaller radius of the waist taper region, that can generate a larger local electric field in the tapered region, thus generating a higher power evanescent field. The high-power evanescent field has the ability to detect subtle changes on the fiber surface, including biomolecules, temperature, pressure, chemical ions, and gases. This results in a higher level of sensitivity and an improved limit of detection (LOD) for the sensor [32,33,34,35]. As a result, optical fiber sensors with TOF structures have numerous applications in a variety of domains [36,37].

The TOF sensor also has many other distinguishing features. Firstly, it is compact and can be flexibly installed for measurement and detection in various environments [38]. Secondly, TOF does not require complex processing and expensive materials, and the preparation cost is relatively low [39]. In addition, TOF processing accuracy is high, and the preparation process is relatively easy to control with high reproducibility. At the same time, TOF has a large surface area at the tapered structure, which can better contact the analyte, thus improving the sensing effect [40]. Furthermore, the TOF sensor design is flexible and can realize different functions of the sensor by changing its angular structure, sensing medium, and sensing layer. The optical fiber processed by pulling the taper expands the abrupt field when light is transmitted in the fiber, increases the contact area with the external environment, significantly improves the sensitivity and response speed of the fiber optic sensors, and shortens the size of the fiber-optic sensor [41,42]. The preparation methods of TOF probes include arc discharge technology [43], laser processing technology [44], chemical etching technology [45], etc. TOF structures are commonly found in the following configurations: single tapered structure [46], nano-tapered fiber [47], grating tapered structure [48], multi-tapered cascade structure [49], taper tip [50], and so on. Internal beam splitting and optical path coupling will occur as a result of the change in fiber geometry. Moreover, the evanescent field near the tapered fiber will cause the light to diffuse into the surrounding environment as well as change the beam splitting and coupling ratio of the tapered fiber. The fiber-optic sensor with a tapered structure of can determine the refractive index (RI), curvature, strain, and other physical quantities of the surrounding environment. In conclusion, based on the characteristics and advantages of TOF sensors, it will provide a broader development space and a new platform for optical fiber sensor research.

Tapered optical fiber-based sensors have gained popularity in various fields such as gas sensing, physical sensing, chemical sensing, and biological sensing in recent years. Shaimerdenova et al. [51] reported a shallow tapered reflection-free fiber optic sensor for cancer biomarker detection. The sensor utilizes magnesium oxide nanoparticle-doped optical fibers and is functionalized using a silylation method to detect the breast cancer biomarker CD44 protein. The results show that the proposed sensor prototype is capable of measuring CD44 protein with remarkably low detection limits and high specificity. Similarly, Ayupova et al. [52] proposed a sensor based on a gold-modified shallow-tapered chirped fiber Bragg grating (FBG) to simultaneously monitor RI and temperature. This paper reports a method based on the fabrication of a shallow fiber taper made with a CO_2_ laser on an inscribed chirped fiber grating. Experimental spectral analysis reveals a pre-taper region that is insensitive to RI and a post-taper region where the reflectivity level varies with RI. The sensitivity of the sensor is 382.83 dB/RIU and 9.893 pm/°C for RI and temperature measurements, respectively, and the sensor is capable of operating at low crosstalk. Due to its dual sensitivity and compact size, the sensor is a promising technology for the implementation of fiber optic biosensors for in situ analysis and long-term monitoring. 

Researchers have also shown interest in TOF sensors that are immobilized with cell layers. Tapered fiber optic sensors attached to *Pseudomonas putida* TVA8 bioluminescent bioreporters were designed by Zajíc et al. [53]. The cell layer was attached to the fiber surface by (3-aminopropyl) triethoxy silane modification. The experimental results demonstrated that the tapered fiber optic sensor based on cell immobilization was capable of toluene detection. A microfiber interferometer-based fiber ring laser was developed for the detection of Listeria monocytogenes by Li et al. [54]. The sensor employs a single mode-tapered no core-single mode structure and is functionalized with a relevant antibody. The erbium-doped fiber amplifier excites the laser used in the sensing system, which reduces the half-height width of the measurement spectrum, thereby greatly increasing the limit of LOD. The stability and specificity of the sensor were proven by testing on actual samples.

In this review, the sensing mechanism and fabrication approaches of TOF sensors are summarized, and the application and development of TOF sensing technology for practical scenarios from physical, chemical, and biological aspects are comprehensively analyzed. The development of TOF fiber-optic sensors has been realized for physical, chemical, and biological applications. In the future, as TOF sensor preparation technology and optical sensing technology continue to develop, the TOF sensor is expected to deepen and expand into more application fields.

## 2. Theoretical Mechanism for Sensing

**Figure 1:**
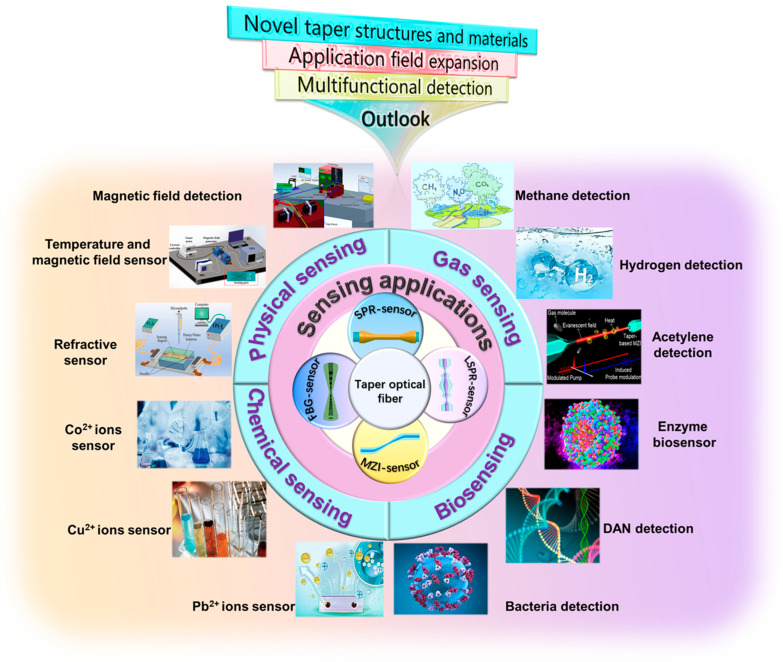
Schematic of the TOF sensors used in different sensing applications [58,59,60,61].

TOF as an optical sensor configuration, because of its simple fabrication, low cost, and good stability, progressively evolved into one of the most extensively used fiber-optic devices [55]. Its sensing approach is based primarily on light propagation characteristics in the tapered area and interaction with the external environment [56]. By changing the radius of the fiber, the original ratio of the fiber core to the cladding is changed. That is mainly used to excite the higher-order modes of light transmission in the fiber [57], allowing the interaction between the optical field inside the fiber and external physical parameters, chemical substances, and biological molecules for sensing purposes. This section will elucidate the fundamental structure of TOF, including the evanescent waves (EWs) sensing theory, the surface plasmon resonance (SPR)/localized surface plasmon resonance (LSPR) sensor mechanism, in-line Mach-Zehnder interferometer (MZI)sensing theory, and the grating principle. Figure 1 depicts the advances in tapered optical fiber sensor structures and those applications.

### 2.1. Basic Structure of TOF

**Figure 2:**
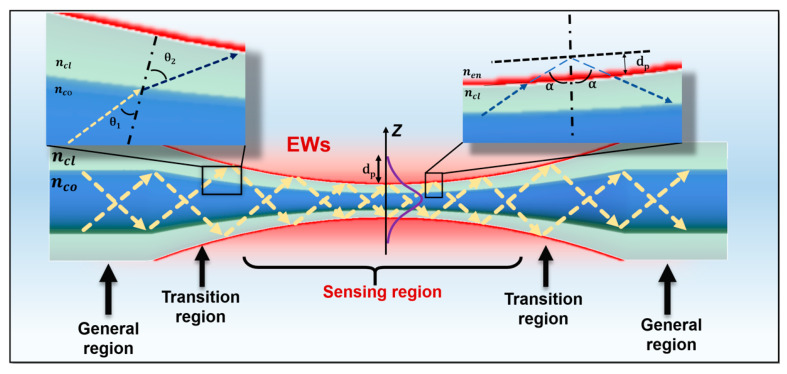
Schematic of basic tapered fiber structure.

Figure 2 illustrates the basic structure of a TOF and how light propagates through the TOF structure. The schematic of the TOF structure is composed of single-mode fiber (SMF) tapered to form three components, consisting of a general fiber region, a transition region, and a tapered waist region (sensing region), in a centrosymmetric structure. The core and cladding diameter of the transition area decreases linearly and slowly, while the sensing region remains a cylindrical shape with a uniform diameter. By controlling the parameters of the tapered structure, such as the diameter and length of the sensing region and the transition region mutation angle, different tapered fibers with different sensing characteristics can be realized for different application situations.

### 2.2. Evanescent Waves Sensing Principle of TOF

Light transmitted through the fiber core follows the principle of total internal reflection (TIR) [13]. In other words, light transmission in optical fibers is governed by Snell’s law, which reveals at the equation level the refraction phenomenon when light propagates from one medium to another, namely [62]:

(1) $$n_{co}sin\theta_{1}=n_{cl}sin\theta_{2}$$

Here $n_{co}$ and $n_{cl}$ are the RI of the incident and transmitted media, respectively. And $\theta_{1}$ and $\theta_{2}$ are the angles of incidence and refraction. In addition, $\theta_{2}$ increases with the increase of $\theta_{1}$. When $\theta_{2}$ is 90°, the refracted light disappears, and the condition of total reflection is satisfied. At this time, the beam is transmitted inside the core, which is known as TIR [63]. According to the TIR principle, when the angle of incidence is greater than the critical angle, the light can be transmitted without loss [64]. However, this is not the case, when the light at the core-cladding interface of the fiber occurs in total reflection, there will also be part of the energy leakage into the cladding, the field strength is very weak, and exponential decay, so the attenuation field is called the evanescent field [65]. The evanescent field is the optical field outside the core, which plays an important role in fiber bending sensing, and different modes of fiber transmission have different evanescent fields. Figure 2 demonstrates that in the general region of the fiber, the light propagation is confined within the fiber core due to the TIR principle. In SMF, the original TIR propagation pattern is changed during the gradual thinning of the transition area, and the evanescent field is greatly enhanced in the thinning cross-section region. In this case, the evanescently departing field formed near the tapered area can be used to enhance the SPR/LSPR effect. In the TOF sensing region, the transmitted light waves are penet into the cladding for a certain distance and then returned to the core [66]. The field through the core interface is referred to as the evanescent field. The propagation direction of the EWs is perpendicular to the fiber axis and decays exponentially [67,68]. The mathematical formula for the field strength of the evanescent field can be expressed as:

(2) $$E=E_{0}\exp\left(-\frac{\delta }{d_{p}}\right)$$

Here δ denotes the distance from the dividing interface and $E_{0}$ denotes the field strength on the dividing surface. The longitudinal distance through the cladding of the EWs, i.e., the penetration depth $d_{p}$, is defined in Equation (3) [69] ($d_{p}$ is also the depth when the energy of the evanescent field is reduced to 1/e of the energy at the interface).

(3) $$d_{p}=\frac{\lambda }{2\pi \sqrt{{{(n}_{cl}sin\alpha )}^{2}-{n_{en}}^{2}}}$$

where λ is the incident light wavelength, α is the angle of incidence of light at the interface between the fiber cladding and the external environment, $n_{cl}$ and $n_{en}$ are the RI of the fiber cladding and the external environment, respectively. 

The $d_{p}$ is dependent on the change in the surrounding RI, so the TOF sensor can detect information about biomolecular analytes by detecting changes in the sensitive RI. The greater the $d_{p}$ of the EWs, the more its energy leakage interacts with the external environment. Therefore, increasing the sensitivity of the sensor can be achieved by increasing the $d_{p}$ of the EWs. The advantage of tapered fiber is that it breaks the inherent TIR transmission mode, has a larger mode field, and wider spectral range, and is more conducive to the excitation of surface EWs. As a sensing element, tapered fiber has a strong evanescent field near its tapered region, that is more susceptible to modulation by the external environment, making it very promising for high-sensitivity measurements, and its small size is also one of its distinctive advantages [70].

### 2.3. SPR/LSPR Sensor Mechanism Based on TOF

The SPR/LSPR phenomenon occurs between the surface of the metal film/nanoparticles (NPs) where the sensor has a specific molecular recognition element and the vacuum/air or liquid medium. When the analyte molecule binds to the enzyme/antibody/aptamer of the sensor, it alters the surface of either the fiber-coated metal film or NPs. This alteration causes a shift in the position of the SPR spectral peak, which is caused by a change in RI on the metal surface [71].

LSPR and SPR are both sensing techniques based on the plasmon resonance principle. Their basic principles can be described by the Maxwell equation [72] and the Kretschmann configuration [73]. Unlike the SPR phenomenon, which utilizes metal film excitation to detect the presence and concentrations of chemicals and biomolecules in analytes, LSPR uses NP excitation to detect the presence and concentrations of chemicals and biomolecules in analytes.

SPR sensing is a technique that uses the surface plasmon resonance phenomenon of metals for sensing. In SPR biosensors, a bio-recognition element is immobilized on the surface of a metal film. When the target analyte binds to the biometric element, it causes a change in the RI of the metal film surface, which changes the resonance angle and the intensity of the reflected light from the SPR phenomenon. By measuring this change in resonance peak and reflected light intensity, the concentration and characteristics of the molecule to be measured can be quantified [74]. With the advantages of fast, sensitive, real-time and label-free detection, SPR biosensors have become advanced and powerful tools for characterizing and quantitatively analyzing biological properties [75]. Although fiber-optic SPR sensors are highly sensitive, the conditions for excitation of SPR are complex and the direct detection of low molecular weight analytes at low concentrations is challenging.

LSPR sensor is a technology that uses the localized plasmon resonance phenomenon of NPs for sensing. In LSPR sensors, the size and shape of the NPs affect the different wavelengths of the absorbed and scattered light, thus influencing the excitation efficiency of the LSPR phenomenon [76]. When a target analyte binds to a ligand on the surface of metal-NPs, it causes a change in the RI around the NPs, resulting in a shift and change in the LSPR spectral resonance peak of the metal-NPs. The detection purpose is achieved by monitoring this change in the position of the resonance peak. The tapered structure is one of the most popular fiber-optic probe structures in the field of LSPR fiber-optic sensing.

SPR/LSPR is an optical phenomenon that occurs in metal thin films/NPs (e.g., gold, silver) [77]. When leaked light from the fiber interacts with free electrons in the metal film/NPs on the fiber surface, the electrons collectively oscillate at a specific frequency, resulting in (localized) SPR. In the case of the LSPR phenomenon, the resonance wavelength of the LSPR spectrum can be expressed as [78]:

(4) $$\lambda_{res}=\lambda_{w}\sqrt{2n_{e}^{2}+1}$$

The wavelength corresponding to the bulk metal plasma frequency is denoted by $\lambda_{w}$, while the RI of the surrounding environment is represented by $n_{e}$. When the RI of the dielectric on the surface of the metal layer is changed, a new resonance of the incident light at another angular frequency occurs. This can be expressed macroscopically as a change in the resonance wavelength. The amount of change in resonance wavelength can be expressed by Equation (5) [79].

(5) $$\Delta \lambda_{res}=S\Delta n_{d}\left[1-\exp(-\frac{2d}{l_{d}})\right]$$

Here S denotes the bulk RI response of NPs, $\Delta n_{d}$ is the amount of change in the surrounding environment’s RI, d represents the effective adsorption layer thickness and $l_{d}$ is the attenuation length of the characteristic electromagnetic field.

In recent years, SPR/LSPR-based fiber optic sensors have made remarkable progress in biosensing, environmental monitoring, chemical analysis, and other fields. On the one hand, as nanomaterials and nanotechnology advance, the sensitivity and selectivity of SPR/LSPR-based sensors make improvements. On the other hand, the multifunctionality of sensors is also realized with the introduction of multiple recognition elements.

### 2.4. In-Line MZI Theory Based on TOF

Fiber-optic MZI is an important interferometric structure in fiber-optic interferometer, which has received a lot of attention due to its optical filtering characteristics, low fabrication cost, and high accuracy. The TOF structure constitutes an inline MZI configuration and is often used for sensing purposes because of its stability, compactness, and high accuracy [38].

In the case of a standard SMF taper, when light is transmitted to the taper region, the bundling ability of the cladding is weakened due to its thinning diameter, and a small portion of the light leaks to the externally of the core, while the higher-order mode in the cladding is excited and transmitted in the fiber at the same time as the fundamental mode in the core. After passing through the taper area, the two light channels have a phase difference, and interference occurs when they are ultimately coupled back into the fiber core. The sensor’s output light intensity can be calculated using the interference principle. From the interference principle, the output light intensity of the sensor can be determined as [80]:

(6) $$I_{o}=I_{1}+I_{2}+2\sqrt{I_{1}I_{2}}cos∆\varphi$$

$I_{1}$ and $I_{2}$ are the light intensities of the higher-order mode in the cladding and the fundamental mode in the core, respectively, and ∆φ is the phase difference generated by the two light paths, which is defined by the mathematical formula [81]:

(7) $$\Delta \varphi =\frac{2\pi {\Delta n}_{eff}L}{\lambda }$$

${\Delta n}_{eff}$ is the effective RI difference between the higher-order mode and the fundamental mode, λ is the wavelength of the incident light, and L is the effective interference length. When external parameters (temperature, pressure, deformation) are inflicted on the fiber, the phase difference between the two modes is induced to change. The equation reveals that a change in ${\Delta n}_{eff}$ or L causes a variation in the phase difference, which affects the position of the wavelength of the interference spectrum. Thus, the external environmental parameters can be determined by monitoring the shifts of the interference spectrum. 

Hence, the mathematical formula for the free spectral range (*FSR*) of the interference spectrum can be approximately defined as [82]:

(8) $$FSR\approx \frac{\lambda^{2}}{\Delta n_{eff}L}$$

The mode interference principle of TOF has the advantages of high sensitivity, fast response time, and high reliability, and is extensively used for measurement and sensing in biological, chemical, and physical fields.

### 2.5. Grating Sensing Principle of TOF

Tapered fiber grating is a novel category of optical waveguide device with a diameter in the micron or nanometer range, which combines the evanescent field transmission characteristics of a tapered fiber with the wavelength-selective optical properties of a fiber grating. The fast response, high reliability and small size are also the factors that have contributed to its popularity. Tapered fiber grating is formed by engraving a uniform FBG or long-period fiber grating (LPFG) on a taper fiber, that has unique spectral and dispersion characteristics [83]. The grating is cost-effective, and a single grating can achieve multiparameter measurements with higher accuracy and simplified sensor construction.

In a tapered fiber grating sensor, reflective gratings are periodically inscribed in the tapered region of the fiber. These gratings split the light beam passing through the fiber into different frequencies and split the reflected beam into different angles. When FBG sensors are subjected to external physical quantities, such as temperature, pressure, strain, etc., this causes a change in the RI of the grating, which in turn causes a change in the wavelength or intensity of the incident light. Therefore, the sensing purpose can be achieved by measuring the wavelength shift or intensity change of the transmitted light.

FBG is a grating structure realized by changing the propagation path of light waves in optical fibers, and its underlying principle is based on Bragg scattering. Interference occurs when a light wave encounters a periodic change with RI in a medium, resulting in reflection or transmission of the light wave at a specific wavelength, which is referred to as Bragg scattering. In fact, the Bragg grating requires certain conditions to be satisfied, namely the Bragg condition [84].

(9) $$\lambda_{b}=2n_{eff}\Lambda_{b}$$

$\lambda_{b}$, $n_{eff}$, and $\Lambda_{b}$ refer to the reflection peak wavelength, effective RI of the medium, and period of the gratings, respectively. When an FBG sensor is affected by external physical parameters, chemical substances or biological molecules in the environment, it will cause a change in the effective RI of the medium, which leads to a shift in $\lambda_{b}$. Therefore, information about various external parameters can be obtained by monitoring changes in $\lambda_{b}$. FBG sensors have advantages such as fast response speed, high reliability, and accuracy. They are widely used for measuring and monitoring physical, chemical, and biological parameters as well as analyzing substances.

Unlike FBG, LPFG core and cladding modes have the same transmission direction, lower insertion loss, and less backward reflection. In accordance with the phase-matching condition, the coupling wavelength for linear polarization mode can be described as [85]:

(10) $$\lambda_{l}=(n_{eff}^{co}-n_{eff}^{cl})\cdot \Lambda_{l}\Lambda_{l}$$

Here, $n_{eff}^{co}$ and $n_{eff}^{cl}$ denote the effective RI of the core and cladding, respectively. $\Lambda_{l}$ represents the period of the LFPG. Previous studies have indicated that LPFG structures exhibit high sensitivity to RI in the environment surrounding the optical fiber. Based on this property, LPFG has been applied in physical, chemical, and biological sensors [86].

Combining FBG with TOF structures, this not only takes advantage of the geometry of TOF structures, but also incorporates the properties of gratings simultaneously [87]. This structure provides a new fabrication strategy for sensor development.

## 3. Fabrication Method of TOF

Different fabrication methods can be used to manufacture TOF depending on the fabrication requirements of the tapered fiber and the limitations of the equipment. The common fabrication methods include arc discharge technology, laser processing technology, and chemical etching technology. The methods and characteristics of these technologies are summarized in this section. 

### 3.1. Arc Discharge Technology

**Figure 3:**
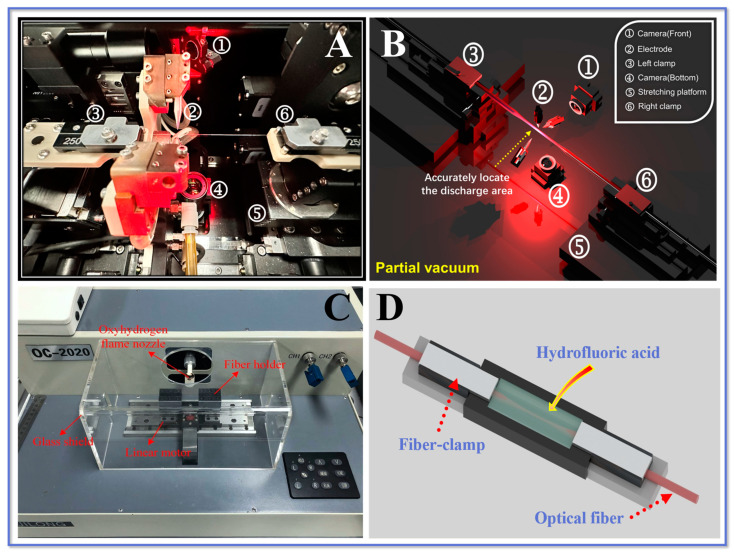
Schematics of TOF fabricating process (**A**). three-electrode system (CMS machine); (**B**). important components of the three-electrode system; (**C**). oxyhydrogen flame heating system. Reprinted with permission from Optics and Laser Technology, Copyright 2020, Elsevier [19]; and (**D**). chemical etching facility.

Arc discharge technology is a processing technique that allows controlled electrodes to periodically heat the fiber layer to a molten state by freely adjusting the discharge power and precisely limiting the discharge time. Compared with the hydroxide flame heating method, the arc discharge technique is able to form a highly uniform high-temperature area around the optical fiber to heat it up, enabling faster softening of the fiber and greatly improving processing efficiency. For instance, the combiner manufacturing system (CMS) machine is used to heat the optical fiber using thermally stabilized plasma technology. The vacuum saturation and the discharge power between the electrodes are adjusted using a program to achieve optimal heating conditions. The important internal components of the CMS are shown in Figure 3A,B, mainly composed of electrodes, fiber clamps, camera, and a movable motor platform. The vacuum module uses the gas pressure principle and a peripheral air pressure pump to maintain a constant semi-vacuum around the fiber. The three electrodes discharge synchronously and flexibly to cover uniformly the maximum thermal zone size of the said fiber. When the fiber reaches the molten condition, the platform motor stretches it under the control of a predetermined program, producing a tapered structure. This method can produce high-quality, well-shaped optical fiber structures in a shorter period of time. 

### 3.2. Laser Processing Technology

The laser processing technique is a typical approach for manufacturing TOFs. The fundamental concept is to employ the laser’s high energy and precision to locally erode the fiber surface for constructing a TOF structure. The laser beam is specifically focused on the fiber surface via a lens, causing the material on the fiber surface to melt or peel, resulting in a reduction in fiber diameter and shape modulation. This technology has applications for fabricating TOF of diverse shapes and diameters because of its superior precision, controllability, and reproducibility. The schematic diagram of TOF manufacturing by laser technology is presented in Figure 3C.

### 3.3. Chemical Etching Technology

Chemical etching is a common method for TOF fabrication, which involves etching the fiber surface through a chemical reaction [88]. The taper angle of the TOF can be controlled by varying the time the fiber is treated in the chemical reagent. The size of the taper angle can affect the efficiency of light transmission in the fiber. The fabrication process has simple equipment and low cost, but it is difficult to control the taper angle and the diameter of TOF precisely. The chemical reaction conditions and operation process need to be strictly controlled to ensure the quality of the fabricated TOF [89]. The schematic of TOF fabrication based on the chemical etching technique is illustrated in Figure 3D.

Overall, each of the above techniques for fabricating TOF has its own advantages and disadvantages, and the most suitable manufacturing method needs to be selected according to the specific application requirements and experimental conditions. Figure 3 presents some process schematics for TOF probe fabricating.

## 4. Signal Demodulation of TOF Sensors 

The advancement of sensing technology has contributed to the rapid development of TOF sensors that are based on signal demodulation. Fiber-based signal demodulation techniques are often used for more intricate sensing systems, such as distributed sensing, interferometer sensing, FBGs sensing, etc. This section briefly describes several commonly used signal demodulation methods and their applications.

### 4.1. Overview of Signal Demodulation Techniques

Signal demodulation is a concept originating from communication systems, i.e., the technique of processing the modulated signal to extract the original information. This technology plays an important role in the field of fiber optic sensing. In fiber optic sensors, signal demodulation technology usually refers to the process of converting the received optical signals into an electrical signal and extracting the sensed physical quantities (e.g., temperature, pressure, and strain) from them. 

Frequently employed techniques for phase demodulation in fiber optic sensing, including phase generation carrier demodulation, 3 × 3 coupler demodulation, and in-phase quadrature demodulation [90]. The PCG demodulation method employs a carrier signal of high frequency that surpasses the bandwidth of the primary signal. This carrier signal can shift the sidebands of the original signal, thereby mitigating the impact of low-frequency noise on the signal. 3 × 3 coupler demodulation technique is widely used in interferometer type fiber optic sensors, such as, MZI, Michelson interferometer, and Fabry Perot interferometer sensors.

Optical time-domain reflectometry (OTDR) and optical frequency-domain reflectometry (OFDR) are common demodulation techniques used in distributed sensing. OTDR and OFDR technologies are also applied in fiber optic FBG-based sensors. A landslide monitoring sensing system based on OTDR is designed and developed on the foundation of bending loss of optical fiber by Zheng and co-authors [91]. Kwon et al. [92] reported a distributed fiber optic temperature sensor with aluminum layer enhancement sensitivity using Rayleigh backscattering spectral shifts of OFDR. The experimental results indicated that the performance of the aluminum-coated fiber sensor was superior to that of conventional single-mode fiber sensing. 

### 4.2. TOF Sensors Based on Signal Demodulation Techniques

In recent years, TOF-based signal modulation and demodulation sensing systems have also proliferated. Liu et al. [93] developed a TOF sensor based on the MZI configuration for seawater temperature, salinity, and pressure. The sensing system uses the sensitivity matrix method as well as machine learning methods to demodulate the signals with cross-sensitivity. Similarly, Yu et al. [94] introduced a TOF-based all-fiber optic sensor for seawater to achieve the monitoring of salinity, temperature, and depth in seawater. The sensor was fabricated by combining conventional optical fibers into a TOF. The sensitivity of salinity and temperature-depth was 1596 pm/‰, 2326 pm/°C, and 169 pm/MPa, respectively.

Yi and colleagues [95] introduced a sensor for measuring RI that utilizes an FPI. Initially, the study employs a femtosecond laser to inscribe a cavity of FPI in a conventional SMF. Subsequently, the taper process is carried out utilizing a CO_2_ laser. Simultaneously, the sensor employs the OFDR technique. The findings of the experiment indicate that the sensor applying the tapered intrinsic FPI configuration achieves a resolution of 2 × 10^−5^.

Cardiac troponin I (cTnI) is crucial for the emergency diagnosis of cardiovascular diseases. Niu et al. [96] proposed for the detection of cTnI-C a fiber-optic integrated chip immunosensor based on a time-delayed dispersive microwave photonic analyzer. In this work, a whispering gallery mode fiber optic probe was manufactured, and the laser output wavelength was used to achieve cTnI-C concentrations detection. The sensing realized a high-resolution demodulation approach based on wavelength demodulation. Eventually, the sensing probe obtained a detection resolution with LOD of 1.2 fg/mL and 0.59 ng/mL, respectively.

## 5. Applications of TOF Sensors 

TOF sensors can monitor temperature, pressure, strain, chemical substances, and RI by utilizing the transmission characteristics of light to measure various parameters of the surrounding environment. This review focuses on the application and research progress of TOF sensors in these fields and summarizes their advantages and development prospects to provide reference and guidance for subsequent research and applications.

### 5.1. Physical Applications of TOF Sensors

**Figure 4:**
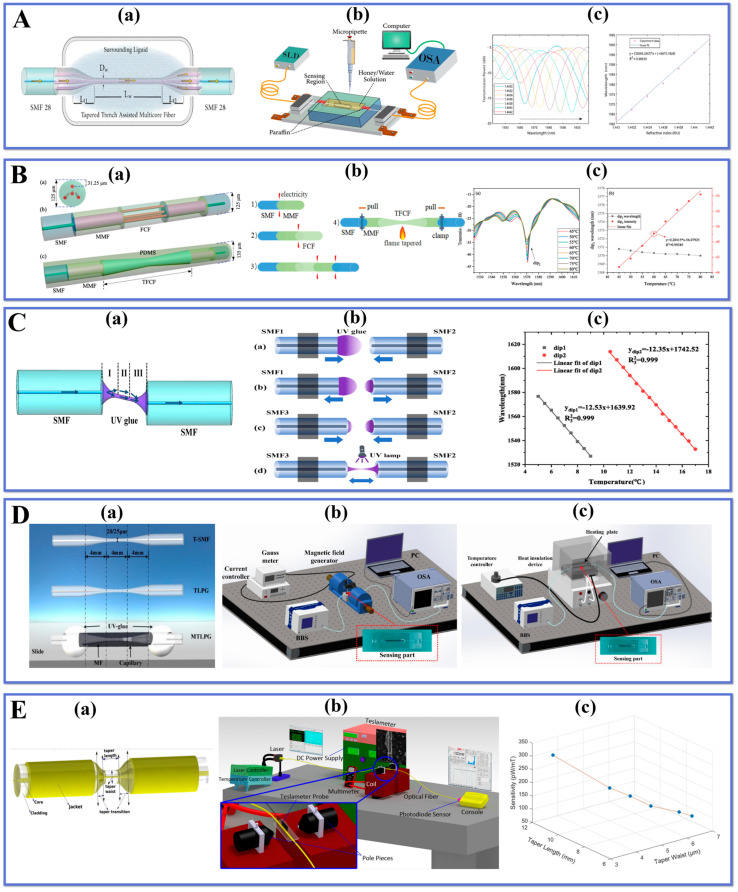
Schematic diagrams of (**A**). (**a**) SMF28-tapered TAMCF–SMF28 structure and (**b**) Experimental setup for SRI sensing, (**c**) measured transmission spectrum and linearity plot results. Reprinted with permission from Optical Fiber Technology, Copyright 2019, Elsevier [58]; schematic diagrams of (**B**). (**a**) FCF cross section, MMF-FCF-MMF structure, T-MFM-F structure coated with PDMS and (**b**) fabrication process of T-MFM-T structure, (**c**) interference spectra of the T-MFM-F sensor, and the relationship between wavelength and temperature. Reprinted with permission from Optics and Lasers in Engineering, Copyright 2023, Elsevier [59]; schematic diagrams of (**C**). (**a**) taper-shaped UV glue structure and (**b**) fabrication process of taper-shaped UV glue, (**c**) the relationship between wavelength and temperature. Reprinted with permission from Optical Fiber Technology, Copyright 2022, Elsevier [60]; schematic diagrams of (**D**). (**a**) preparation process MTLPG structure, (**b**) experimental setup of magnetic field sensor, (**c**) temperature sensing device. Reprinted with permission from Optical Fiber Technology, Copyright 2021, Elsevier [97]; schematic diagrams of (**E**). (**a**) TOF-based structure and (**b**) experimental setup of magnetic field sensor, (**c**) sensitivity in relation to TOF ‘s length and diameter. Reprinted with permission from Optical Fiber Technology, Copyright 2022, Elsevier [98].

A surrounding RI (SRI) sensor on the basis of tapered channel-assisted multicore fiber (TAMCF) was first presented by AL-Mashhadani et al. [58]. The sensor with SMF-TAMCF-SMF structure was fabricated by connecting a section of TAMCF with two SMFs through an arc discharge technique, and the schematic of the structure is displayed in Figure 4A(a). TAMCF has seven cores, each of which is surrounded by a separate cladding and groove layer. Due to its unique fiber optic structure, it has multiple applications in various fields of sensing. Figure 4A(b) shows the experimental setup for sensing. The tapered sensor’s sensitivity was evaluated in various SRI ranges using a variety of honey/water mixtures as SRI standard samples. In the RI range of 1.4430–1.4442, the sensitivity is 35089.28 nm/RIU, which is much higher than other MCF-based RI sensors. The SRI sensitivity increases as the TAMCF waist length expands. As shown in Figure 4A(c), the fabricated tapered sensor has high SRI sensitivity and good linearity, which can flexibly and precisely detect small changes in SRI. The sensor’s advantages include high sensitivity, ease of preparation, and versatility. This paper describes a straightforward and efficient way for creating a high-performance SRI sensor based on TAMCF, that offers potential for development in the biosensing and environmental monitoring domains.

Shao et al. [59] developed a new fiber sensor that can measure both temperature and magnetic field using micro-tapered long-period grating (MTLPG) and magnetic fluid (MF). The TLPG is created by periodic exposure with a CO_2_ laser, combining the advantages of TOF and LPG. Figure 4B(a) shows the probe fabrication process, where SMF is tapered to a diameter of 20 µm by a fusion splicer to obtain T-SMF. A slenderer micro-taper is formed in the waist region of T-SMF due to differences in motor movement speed and softening of the fiber. Then, the CO_2_ laser moves along its axis for periodic exposure to achieve the target structure. The MTLPG comprises a tapered SMF wrapped around its surface with an MF layer whose RI varies with temperature and magnetic field affecting the transmission spectrum. Simultaneous measurement of temperature and magnetic field can be achieved by monitoring the wavelength shift of two resonance peaks in the transmission spectrum as shown in Figure 4B(b,c), respectively. The results show that MTLPG has high sensitivity, fast response time, and good stability at low cost making it ideal for detecting temperature (−0.52 nm/°C sensitivity from 25–75 °C range) or magnetic fields (23.72 nm/mT sensitivity from 8–16 mT range) under various circumstances as a dual-parameter TOF sensor.

Bakhshi et al. [60] proposed an MF detection method based on a TOF sensor, which utilizes a composite coating of graphene oxide (GO) and iron oxide (Fe_3_O_4_) to enhance the magnetic-optical effect. Figure 4C(a) shows the schematic of the TOF structure, which is formed by locally heating the optical fiber to make it thinner during the tapered process to form the TOF. By adjusting the heating power and moving speed, the temperature and position of the heating can be precisely controlled to achieve uniform thinning of the fiber-optic. Figure 4C(b,c) shows the diagram of the device for measuring the magnetic field, and the sensor is highly linear in the magnetic field range of 1–60 mT, respectively. Furthermore, the effect of parameters such as TOF length and diameter on sensor performance was investigated. After optimizing the analysis, it was discovered that the sensor’s sensitivity reached 320 pW/mT when the TOF length was 11 mm and the diameter was 3.3 m. This sensor offers a cost-effective solution to achieve high-performance magnetic field detection utilizing TOF.

Chen et al. [97] presented a polydimethylsiloxane (PDMS) fiber optic sensor based on the taper-MMF-FCF-MMF (T-MFM-F) structure to be capable of accurate temperature measurement. The schematic diagram and fabrication process of T-MFM-F are shown in Figure 4D(a,b). In the field of temperature sensing, PDMS has superior thermal and optical properties compared to other materials, which can enhance its sensitivity to temperature. By applying PDMS to TOF, not only the sensitivity of the sensor is significantly improved, but also the structural strength of the fiber is improved. The results are shown in Figure 4D(c). The sensor has high sensitivity and linearity in the temperature range of 45–80 °C with a sensitivity of 0.046 dB/°C. It also has good repeatability and stability. The sensor has the advantages of simple structure, easy fabrication, and compact size, and can be used in various temperature measurement and monitoring situations.

Liu et al. [98] developed a highly sensitive temperature sensor based on MZI interference with asymmetric tapered UV light irradiation. The sensor utilizes the high RI and low loss of UV optical fiber and the interference effect of asymmetric tapered structure to prepare a special shape of optical fiber sensor and achieve temperature measurement by measuring the interference signal of the sensor at different temperatures. The probe structure consists of two SMFs and an asymmetric tapered UV adhesive connection, and its schematic diagram and fabrication process are shown in Figure 4E(a,b). Experimental results show that the sensor has a temperature sensitivity of 11 nm/°C, and can continuously monitor temperature changes in the response range of 5 °C to 17 °C. Therefore, it can be used for high-precision temperature measurement and sensing applications and has promising applications in the field of temperature measurement.

Li et al. [99] proposed a TOF sensor that simultaneously measures the liquid level as well as the local temperature distribution. The sensor exploits the different responses of various liquid levels and temperatures to the spectra, thus achieving the simultaneous measurement of two physical parameters. The sensitivity of the liquid level and temperature sensing is 0.106 dB/mm and 0.029 dB/°C, 35 pm/°C, respectively. Table 1 summarizes the applications of TOF sensors for physical parameters in recent years.

### 5.2. Chemical Applications of TOF Sensors

**Figure 5:**
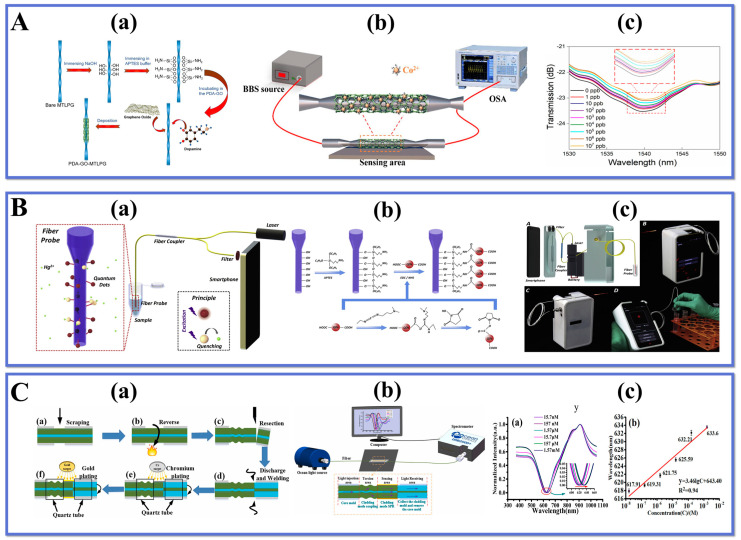
Schematic diagram of (**A**). (**a**) the PDA-GO-MTLPFG functionalization process and (**b**) experimental setup of Co^2^+ sensing, (**c**) transmission spectra GO-MTLPFG for Co^2^+ sensing. Reprinted with permission from Optical Fiber Technology, Copyright 2022, Elsevier [110]; schematic diagram of (**B**). (**a**) SOFFS Principle and (**b**) QD functionalization process, (**c**) SOFFS hardware design. Reprinted with permission from Sensors and Actuators B: Chemical, Copyright 2019, Elsevier [111]; schematic diagram of (**C**). (**a**) fabrication process of twisted fiber structure and (**b**) RI sensing test equipment, (**c**) SPR resonance spectrum and relationship of Cu^2^+ concentration and resonance wavelength. Reprinted with permission from Optik, Copyright 2022, Elsevier [112].

Kang et al. [110] reported a dopamine-functionalized GO for the preparation of a highly sensitive LPFG chemosensor with application to the detection of cobalt ions. The study was conducted by modifying the GO with dopamine compounds, resulting in a homogeneous and stable coating of GO on the probe, which provides binding sites for cobalt ions. The surface functionalization of the probe is depicted in Figure 5A(a). Micro-tapered LPFG was fabricated by CO_2_ laser using single-mode fiber. The micro-tapered LPFG has a diameter of 110 μm, which has a higher mechanical strength than the small diameter TOF and is well suited for sensing experiments in complex environments. 

The experimental setup for Co^2+^ ion detection is shown in Figure 5A(b), and the spectral detection of cobalt ions in the concentration range of 1 ppb–10^7^ ppb was performed. The results are illustrated in Figure 5A(c). The maximum resonance wavelength shift was 0.85 nm with increasing cobalt ion concentration. The high sensitivity of 2.4 × 10^−3^ dB∙ppb^−1^ was obtained at a PDA concentration of 0.05 g∙L^−1^ by using a PDA-GO functionalized probe. The proposed PDA-GO functionalized sensing probe has excellent performance for efficient detection in low-concentration cobalt ion environments, providing a new platform in the field of biological and chemical sensing.

Mercury ion (Hg^2+^) is a toxic heavy metal that has serious harmful effects on humans and environment. Therefore, fast, accurate, and convenient sensors are needed for the quantitative and qualitative detection of Hg^2+^. Liu et al. [111] designed a smartphone-based optical fiber fluorescence sensor (SOFFS) for remote and on-site quantitative detection of Hg^2+^ in water. The schematic of the sensor experimental apparatus is demonstrated in Figure 5B(a). According to the procedures in Figure 5B(b), quantum dots are modified on the fiber surface by layer-by-layer reaction. SOFFS utilizes a fluorescent probe to achieve selective recognition and fluorescence burst of Hg^2+^ by specific ligand reaction with Hg^2+^. 

In addition, the study designed a smartphone application as shown in Figure 5B(c) for automatic and accurate quantification of fluorescence burst and thus measurement of Hg^2+^ concentration. The optimized combinatorial tapered fiber optic probe in SOFFS was able to effectively detect Hg^2+^ in the detection range of 1–1000 nM with the LOD of 1 nM. The fiber optic probe was fabricated with good quality and high reproducibility, and the results demonstrate that SOFFS has good selectivity in Hg^2+^ sensing. In addition, SOFFS is a potential tool for small size portable, low cost and high-efficiency water quality monitoring. It can measure Hg^2+^ quickly and with excellent selectivity, high accuracy and sensitivity, proving its potential value in environmental monitoring.

Wei et al. [112] developed a new sensor for detecting copper ions (Cu^2+^) using twisted optical fiber SPR technology. The degree and angle of the fiber twist were adjusted to modulate the SPR response, as shown in Figure 5C(a). A nano-metal film was then coated on the cladding’s external surface to excite the fiber SPR effect at the interface between the cladding and sensing film. To detect Cu^2+^ concentration, chitosan (CS) and polyacrylic acid (PAA) were modified on the probe surface alternately through electrostatic adsorption. The sensing test device is depicted in Figure 5C(b). The sensor uses a polymer containing amino and carboxyl groups as a sensitive layer that can effectively adsorb copper ions, causing a red shift in the SPR spectrum, as illustrated in Figure 5C(c). The resonance wavelength shifts with increasing Cu^2+^ concentration within the 15.7–1.57 mM range with detection sensitivity of Cu^2+^ being 3.46 nm/lgC and LOD being 10.1 nM. The twisted SPR sensor based on single-mode fiber has simple fabrication, stable structure, high selectivity making it suitable for environmental and biological fields for detecting Cu^2+^.

Wang et al. [113] proposed a tapered interferometer sensor for detecting lead ion (Pb^2+^) concentrations using poly(dopamine-maleic acid) (PDA-MA) functional film enhancement. The microfiber was constructed by heating and uniformly stretching the SMF, which reduced the fiber diameter from 125 μm to 10 μm, resulting in a stable microfiber interferometer. PDA is an emerging material used for molecular immobilization and electrochemical sensing due to its abundant reactive groups that can self-polymerize on various surfaces to form adhesive films. The conical structure and ultra-fine diameter of this interferometer make it highly sensitive to changes in RI caused by the adsorption of carboxyl groups with lead ions, resulting in a macroscopic shift of the wavelength in the interference spectrum. Experimental results show that the sensor can detect lead ion concentration within a range of 10^−14^–10^−6^ M with high sensitivity (1.85 × 108 nm/M) and low LOD (0.1678 ppb). This simple and inexpensive sensor exhibits good stability and specificity, indicating feasibility for developing fiber-optic micrometer sensors with high accuracy for optical environmental pollution research purposes. Figure 5C(a,b) present schematics of the process of PDA-MA functionalization and experimental setup for Pb^2+^ concentration detection while Figure 5C(c) shows the results obtained from experiments conducted on this sensor design.

**Figure 6:**
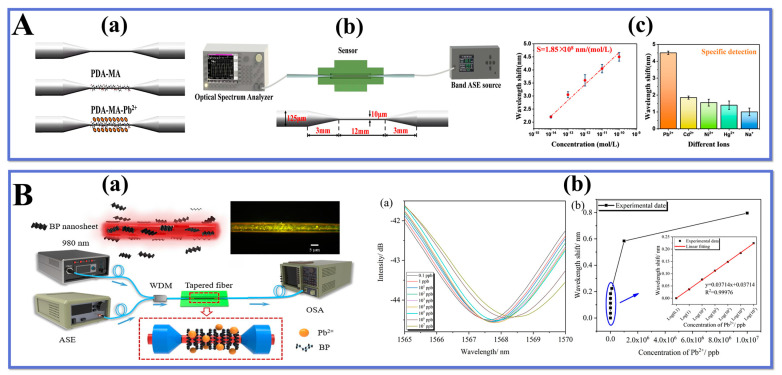
Schematic diagrams of (**A**). (**a**) PDA-MA film functionalized microfiber sensor and (**b**) experimental setup of Pb^2+^ sensing, (**c**) linear response of the probe to Pb^2+,^ and specific detection results. Reprinted with permission from Optics and Laser Technology, Copyright 2023, Elsevier [113]; schematic diagrams of (**B**). (**a**) BP integrated tapered fiber sensor equipment, (**b**) measured transmission spectrum and linearity plot results. Reprinted with permission from Optical Fiber Technology, Copyright 2021, Elsevier [115].

Teng et al. constructed a high-performance fiber-optic sensor species for online detection of Pb^2+^ using black phosphorus and TOF structure. The TOF sensor with a high surface-enhanced Raman scattering (SERS) effect can achieve rapid detection of Pb^2+^. Black phosphorus, a novel two-dimensional material with excellent optical and electronic properties, can be used as a SERS active substrate to enhance the detection capability of the TOF. The experimental apparatus is shown in Figure 6B(a). Pb^2+^ is electrostatically adsorbed onto the BP surface to form adsorption complexes. The accumulated complexes change the RI around the TOF, resulting in a shift in the interference wavelength. The result Figure 6B(b) reflects a significant rightward shift of the spectrum at concentrations from 0.1 to 104 ppb with a shift of 0.184 nm. And Figure 6B(c) illustrates the fitted plot of wavelength drift and Pb^2+^ concentration change, with a sensitivity of 0.03714 nm/ppb and LOD of 0.0206 ppb for the Pb^2+^ solution concentration range of 0.1 to 105 ppb. Through multiple tests, it can be demonstrated that the sensor has good stability and specificity, with the advantages of high sensitivity and LOD. It provides a new approach to environmental safety and water quality detection.

An interferometric TOF sensor for the detection of ferric ions was reported by Yap et al. [114]. The sensor was functionalized with nitrogen and sulfur co-doped carbon dots and exhibited detection performance comparable to that of conventional sensors. The experimental results suggested that the sensitivity of Fe^3+^ detection was 0.0061 nm/(μg/L) and the detection limit was 0.77 μg/L in the linear range of 0–300 μg/L. The applications of TOF sensors for chemical substances in recent years are collected in Table 2.

### 5.3. TOF-Based Gas Sensors

**Figure 7:**
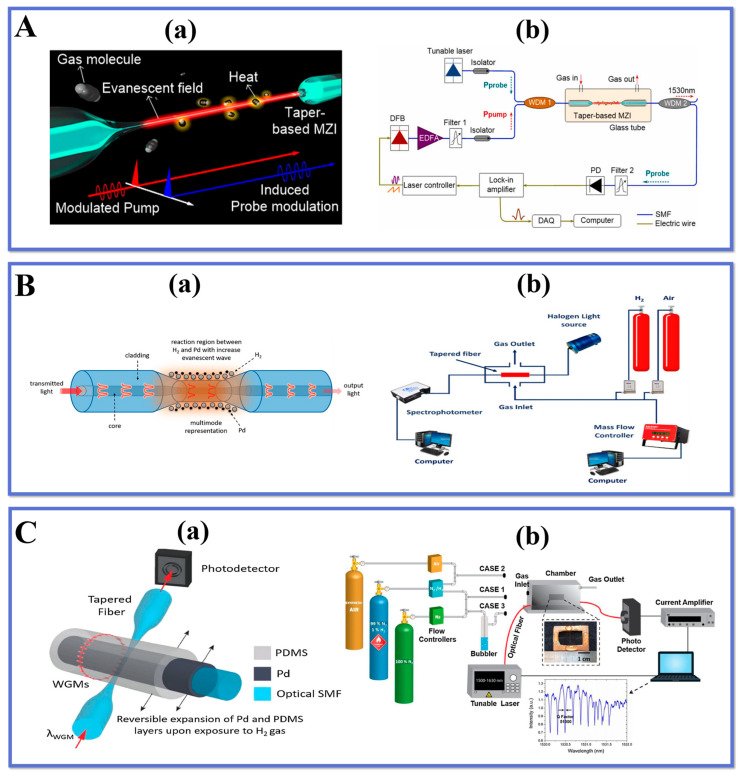
Schematic diagram of (**A**). (**a**) DTP-enhanced PTI gas detection principle, (**b**) experimental setup of PT gas sensing. Reprinted with permission from Sensors and Actuators B: Chemical, Copyright 2023, Elsevier [61]; (**B**). (**a**) palladium and hydrogen detection principle, (**b**) experimental setup of H_2_ sensor, Reprinted with permission from Materials Science and Engineering: B, Copyright 2023, Elsevier [61]; (**C**). (**a**) H_2_ sensing mechanism, (**b**) experimental setup of H_2_ sensor. Reprinted with permission from Sensors and Actuators B: Chemical, Copyright 2020, Elsevier [124].

Tan et al. [61] proposed a dispersion turning point (DTP) -enhanced photothermal interference acetylene gas sensor based on a tapered micro-interferometer. The sensor utilizes the high sensitivity and high-temperature stability near the DTP to achieve accurate measurement of the gas absorption spectrum. The structure of the conical interferometer is shown in Figure 7A(a). The sensing region features a strong evanescent field, which serves as a platform for light-gas interaction. The gas detection device is shown in Figure 7A(b), which works by passing a modulated laser beam through a fiber-optic micro-interferometer, where a portion of the light is absorbed by the gas, resulting in a photothermal effect that causes a phase change in the fiber-optic micro-interferometer. By detecting the output signal of the fiber micro-interferometer, the absorption intensity and concentration of the gas can be obtained. The design was achieved by using a microfiber interferometer with a length of 3 mm and a diameter of 2.29 μm for acetylene measurements, and a lower limit of acetylene detection of 965 ppb was achieved experimentally. By optimizing the design of the tapered interferometer, the sensitivity of the sensor detection will be greatly improved by combining a thinner waist diameter and a longer interaction length. The gas sensor has the advantages of high sensitivity, fast response, and strong resistance to environmental interference, and has a broad development prospect in important applications such as chemical or biochemical sensing and environmental monitoring.

Alkhabet et al. [123] developed a fiber optic H_2_ sensor using TOF coated with palladium NPs, which can safely and efficiently detect the concentration of H_2_ at room temperature. The sensing mechanism is shown in Figure 7B(a), using a multimode optical fiber as the sensing channel, enhancing the evanescent field in the fiber by tapered treatment (20 μm taper waist), and then coating the TOF with palladium NPs by a drop coating method. Palladium is a metal with catalytic properties that can undergo adsorption and desorption reactions with hydrogen gas, thereby altering its resistance and RI. The experimental setup is shown in Figure 7B(b), where the sensor performance developed at room temperature was evaluated through various concentrations of H_2_ gas. The evaluation showed that the Pd-coated sensor based on Pd had a 63% change in absorbance response when exposed to 2.00% H_2_ in synthetic air. The prepared sensor showed good selectivity for H_2_ and did not respond to other gases such as ammonia (NH_3_) and methane (CH_4_). The experimental results are exhibited in Figure 7B(c). A simple, efficient, and reproducible method for the detection of H_2_ is demonstrated. The Pd-coated tapered fiber shows superior H_2_ sensing at low temperatures compared to other conventional H_2_ sensors. Overall, the developed sensor shows excellent sensitivity, selectivity, and stability with promising applications.

Bavili et al. [124] designed a novel fiber-optic hydrogen (H_2_) sensor based on a self-assembled polymer micro-cylindrical ring resonator (PMRR). The H_2_ sensing mechanism is shown in Figure 7C(a). This PMRR is constructed by coating a hydrogen-sensitive composite material Pd-WO3 on SMF and an organic polymer material PDMS with a significant heat responsiveness. The experimental devices for H_2_ detection are displayed in Figure 7C(b), where the spectral shift of the optical resonance is monitored by studying the optical response of the sensor at different H_2_ concentrations. This spectral shift includes changes in resonant peak frequency and intensity for detection purposes. The results of the experiments revealed that the sensor sensitivity was 140 pm/% H_2_ and LOD was 60 ppm when tested in the range of 1% H_2_ concentration, indicating that the sensor exhibited excellent sensitivity and selectivity in a low concentration H_2_ environment. This confirms that The sensor has potential applications in the detection of H_2_. and can provide an effective solution for applications such as hydrogen leak monitoring and H_2_ energy system safety monitoring.

**Figure 8:**
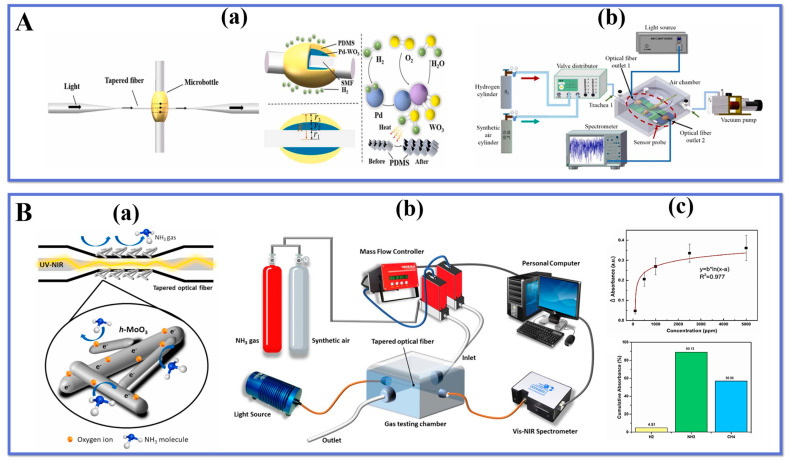
Schematic diagram of (**A**). (**a**) sensor probe and sensing mechanism, (**b**) H_2_ measurement system. Reprinted with permission from Sensors and Actuators B: Chemical, Copyright 2023, Elsevier [125]; Schematic diagram of (**B**). (**a**) NH_3_ sensing mechanism and (**b**) gas measurement system, (**c**) sensitivity curve of A2 for NH_3_ and selectivity test results. Reprinted with permission from Ceramics International, Copyright 2021, Elsevier [135].

Zhang et al. [125] proposed an H_2_ sensor on the basis of a self-assembled micro-vial type resonator, whose structure and principle are shown in Figure 8A(a). The sensor utilizes an SMF coated with a layer of hydrogen-sensitive Pd-WO_3_ composite and a layer of PDMS, an organic polymer material with significant thermal response, to prepare a self-assembled micro-vial structure. The experimental system is depicted in Figure 8A(b), and the whispering gallery mode (WGM) is excited by coupling the TOF with the self-assembled micro-vials. When hydrogen is applied to the WGM resonator, hydrogen molecules penetrate the PDMS and undertake a redox reaction with Pd-WO_3_. After absorbing the reaction heat, the volume and RI of the PDMS change, resulting in a wavelength shift of the WGM resonator. 

The prompt and precise identification of noxious and perilous gases in the surroundings is a crucial approach to avert incidents of poisoning and leakage. Wang et al. [126] utilized a flame fusion biconical taper technique to fabricate a gas-sensitive interferometer with a tapered microfiber structure that was coated with WO_3_ nanorods. The surface of the sensor is modified with WO_3_ nanorods that exhibit the ability to absorb ammonia molecules and facilitate charge transfer, resulting in a consequential shift in the transmission spectrum. 

The experimental results indicate that the sensor exhibits elevated levels of sensitivity and selectivity towards ammonia gas. The study witnessed a spectral shift of 16.23 nm in the WO_3_ nanorod-coated sensor across a range of ammonia concentrations from 0 to 11,640 ppm. The sensor exhibits favorable characteristics in terms of its repeatability and selectivity, thereby indicating the promising potential for a diverse range of applications. The applications of TOF sensors for gases in the last years are collected in Table 3.

As a result of measuring the shift in the resonance wavelength, a low-cost hydrogen sensor is realized. According to the experimental results, the sensor has a maximum sensitivity of −3.091 nm/%. The sensor is resistant to environmental humidity and temperature fluctuations. Furthermore, the sensor is resistant to changes in humidity and temperature. It can also measure other gases using other sensitive materials, which has a wide range of applications in the field of biochemical sensing. Chua et al. [135] reported a method to grow h-MoO_3_ nanorods on the surface of TOF using chemical bath deposition for the fabrication of ammonia gas sensors. The sensing mechanism is shown in Figure 8B(b). The h-MoO_3_ is a semiconductor material with a layered structure and space charge layer effect, which can effectively adsorb and desorb ammonia molecules due to its high specific surface area and excellent optical properties, leading to changes in its resistivity and RI, which affect the optical fiber output light intensity.

The test apparatus is shown in Figure 8B(b), where the h-MoO_3_ coated on the TOF probe is directly connected to the SMA fiber and positioned in a sealed custom gas chamber. The sensing results demonstrated a stable response in the range of 100–5000 ppm. The response time and recovery time were 210 s and 241 s, respectively, at an ammonia concentration of 500 ppm. the optical fiber showed good selectivity for ammonia with an LOD of 11 ppm. the sensor exhibited high sensitivity and fast response to ammonia at room temperature with good stability and repeatability. It provides an effective way to develop a new fiber-optic ammonia sensor.

### 5.4. TOF-Based Biosensors

**Figure 9:**
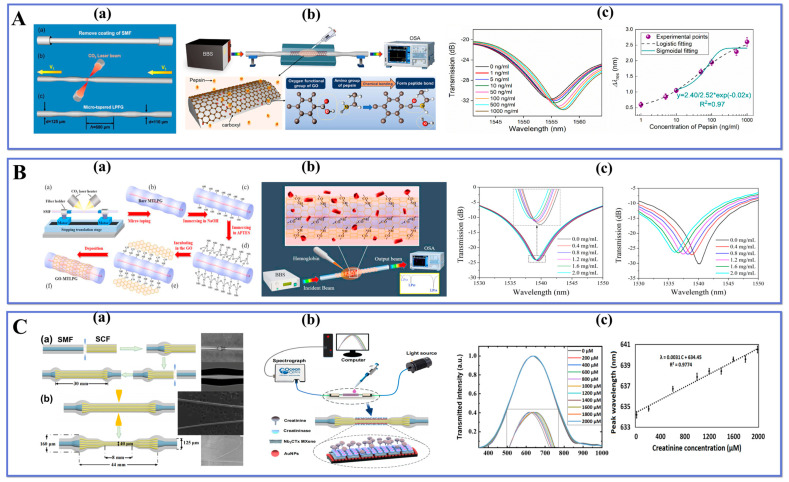
Schematic diagram of (**A**). (**a**) MTLPFG fabrication process and (**b**) measurement system and sensing mechanism, (**c**) measured transmission spectrum results. Reprinted with permission from Sensors and Actuators Reports, Copyright 2023, Elsevier [136]; schematic diagram of (**B**). (**a**) GO nanosheets functionalization process and (**b**) measurement system, (**c**) measured transmission spectrum results. Reprinted with permission from Optical Materials, Copyright 2020, Elsevier [137]; Schematic diagram of (**C**). (**a**) CTC fabrication process and (**b**) measurement system, (**c**) measured transmission spectrum and linearity plot results. Reprinted with permission from Optics Express, Copyright 2022, Elsevier [138].

Kang et al. [136] presented a micro-tapered long-period fiber grating (MTLPFG) pepsin sensor based on GO. GO has excellent biocompatibility and selectivity, and it has a wide application potential in the field of biosensing. As shown in Figure 9A(a), the MTLPFG sensor is processed by CO_2_ laser heating technology, using computer-controlled parameters such as CO_2_ laser power, taper period, and fiber movement speed to control the taper process. The pepsin detection device and sensing principle of the sensor are illustrated in Figure 9A(b). In the experiment, the grating was immersed in different concentrations of sample solutions and the transmission spectra were monitored in real-time. Figure 9A(c) illustrates the transmission spectra and resonance wavelength response results of GO-MTLPFG at different pepsin concentrations. The results indicated that the sensor exhibited high sensitivity in the detection range of 1–1000 ng/mL and the detection limit reached 25.79 ng/mL. In addition, the GO-MTLPFG sensor has good stability and reusability, which offers a new strategy for biosensing applications.

Wang et al. [137] proposed GO nanosheets functionalized with MTLPG-sensitive optical biosensor for hemoglobin detection. In the preparation of the sensor, GO nanosheets were deposited on the probe to form GO-MTLPG by chemical bonding, and the process is displayed in Figure 9B(a). LPG is very sensitive to temperature changes, and the experimental environment was controlled at a constant temperature (23 °C) to avoid experimental accuracy errors caused by temperature differences. The experimental measurement device of the sensor is shown in Figure 9B(b), which meets the requirement of detecting human hemoglobin concentration by monitoring different concentrations of hemoglobin samples. The detection of hemoglobin is achieved when the hemoglobin molecule interacts with the probe surface, resulting in a shift of the resonance wavelength. The experimental results are shown in Figure 9B(c), where the sensor exhibits the blue-shift and a decrease in resonance intensity as the concentration of the hemoglobin sample increases. The sensitivity of the sensor was -2 nm/(mg/mL) and the lowest LOD was 0.02 mg/mL. The GO-MTLPG bioprobe proposed here does not require labeling and can be detected in real-time. It exhibits excellent performance, including high sensitivity, stability, and reusability. This makes it a valuable tool for biomedical research and clinical diagnosis.

Li et al. [138] successfully designed a convex fiber-tapered seven core fiber-convex fiber (CTC) fiber optic LSPR creatinine biosensor with a compact structure and high sensitivity by combining a heterocore fiber with a tapered probe structure. During the processing, CTC is mainly supported by two powerful processing equipment (i.e., FSM and CMS). The FSM electrode discharge process allows the fiber to reach a molten state at a high temperature and form a convex structure under thrust. Under the unique CMS three-electrode heating, the ideal tapered structure is formed by the programmed precise control and bi-directional pulling taper mode, and the CTC structure fabrication process is shown in Figure 9C(a). Two-dimensional nanomaterials (niobium carbide MXene) and The experiments used particular creatinine enzyme functionalized sensing probes, and the experimental setup is shown in Figure 9C(b). The CTC probe was immersed in the reaction cell and the LSPR response results were recorded for different creatinine sample solutions. Figure 9C(c) exhibits the results, in which the wavelength of the resonance peak was evenly red-shifted with increasing concentration in the linear range of 0–2000 μM. The developed sensor’s sensitivity and LOD were 3.1 pm/μM and 86.12 μM, respectively. Moreover, a comprehensive and systematic analysis of the reusability, reproducibility, stability, and selectivity of the sensor probe was carried out and satisfactory results were obtained. It was confirmed that the developed LSPR biosensor has a wide application potential in food safety detection and aquaculture.

**Figure 10:**
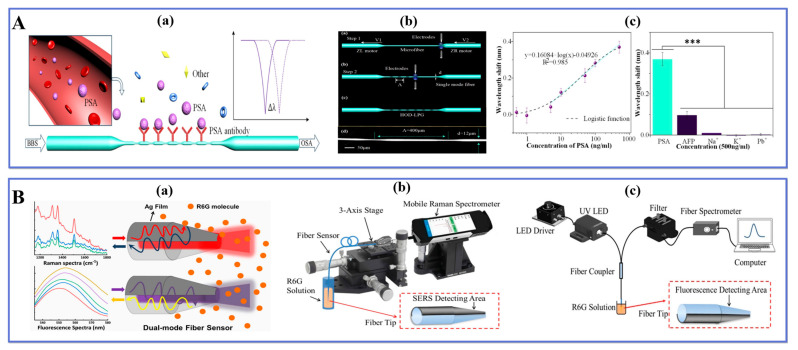
(**A**). Schematic of (**a**) the biosensing process, (**b**) tapering SMF to a microfiber fabrication process, and (**c**) linear response of the probe to PSA and specific detection results. Statistically significant differences, with the *** indicating *p* < 0.01. Reprinted with permission from Optics Express, Copyright 2020, Optica [139]; (**B**). Schematic of (**a**) the dual-mode optical fiber sensor, (**b**) the experimental setup for SERS detection, and (**c**) experimental setup for fluorescence molecules detection. Reprinted with permission from Spectrochimica Acta Part A: Molecular and Biomolecular Spectroscopy, Copyright 2023, Elsevier [140].

The development of new LPF systems with increased sensitivity and stability is critical for practical applications. Xiao et al. [139] demonstrated the use of a high-order diffraction long-period grating (HOD-LPG) for prostate-specific antigen (PSA) detection. Figure 10A(a) shows how the HOD-LPG is biofunctionalized via a multitude of biochemical processes that allow for the detection of minor changes produced by selective recognition of PSA antigens under buffered circumstances. Figure 10A(b) depicts the schematic of HOD-LPG fabrication, which can be separated into two steps: the tapering step and the point-to-point grating inscription. The SMF is tapered into a tapered structure using an FSM machine. This step consists of fixing the fiber on a fixture and moving it along the fiber axis at a certain motor-driven speed. By controlling the arc discharge power and motor-driven speed, a specific ultra-fine tapered fiber structure can be obtained. In the second fabrication step, a portion of the TOF is tapered periodically by setting the grating pitch for point-to-point arc discharge. The experimental results are shown in Figure 10A(c), which exhibits a linear response in the concentration range of 5–500 ng/mL, reaching the LOD of 9.9 ng/mL. The proposed sensor, with its established high repeatability and great specificity, provides a potential tool for the early detection of prostate cancer.

Li et al. [140] proposed a dual-mode fiber optic sensor that utilizes surface-enhanced Raman scattering (SERS) and fluorescence detection in an optical fiber, as illustrated in Figure 10B(a). The sensor was fabricated through two steps: taper processing and Ag coating treatment, with half of the fiber tip surface coated with AgNPs. Initially, a certain length of multimode fiber is processed into a taper and then cut into two tips using a set program that allows good control over the shape parameters of the fiber tip, including tapered arc power and stretching speed. Subsequently, the TOF tip side was fixed with a mold and coated in a high vacuum magnetron sputter coating system to enhance and detect the Raman signal on the coated part while detecting the fluorescence spectrum on the uncoated part. Figure 10B(b,c) show SERS detection test results for Rhodamine 6G aqueous solution concentrations ranging from 0.1–1000 μM along with fluorescence molecular detection devices respectively. The LOD for the SERS detection test was found to be 0.2 µM whereas it was measured at 2.25 µM for fluorescence spectrum within this concentration range. The quantitative identification of target molecules was reliably obtained by combining Raman spectral features and fluorescence intensity since both modes’ detection ranges were well matched. This simple-to-fabricate endoscopic liquid biopsy has excellent performance characteristics making it easy to operate while providing reliable results; hence it holds great application prospects in disease diagnosis/prevention via in vivo detection techniques.

Kamil and colleagues [141] developed a TOF-based sensor that was biofunctionalized and integrated with polyamidoamine (PAMAM) dendrimer. The applications of TOF sensors for biomolecules in recent years are collected in Table 4.

The purpose of this sensor was to detect the presence of dengue E protein. The TOF generated the evanescent field that is very sensitive to changes in the external medium, while the integration of PAMAM increases the adhesion of biorecognition molecules that are complementary to the target protein. Consequently, a greater number of active sites are generated in the tapered region, facilitating the absorption of the DENV II E protein. The sensor exhibited a resolution of 19.53 nm/nM and a detection limit of 1 pM. The reaction constant $K_{d}$ was determined to be 1.02 × 10^−10^ M.

## 6. Novel TOF Structure

**Figure 11:**
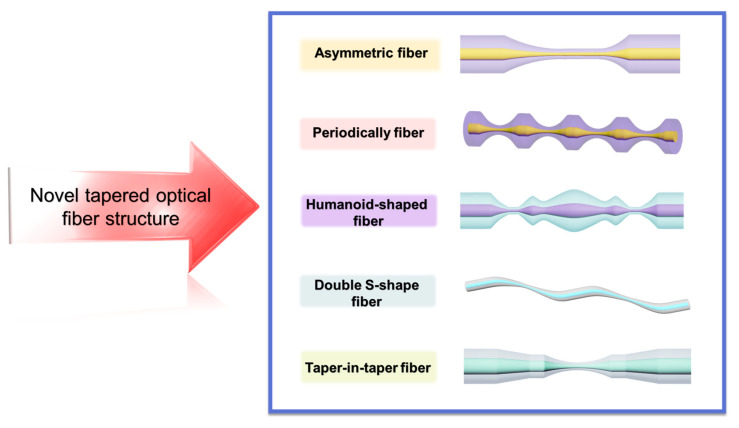
Schematic diagram of innovative TOF structures in recent years.

**Figure 12:**
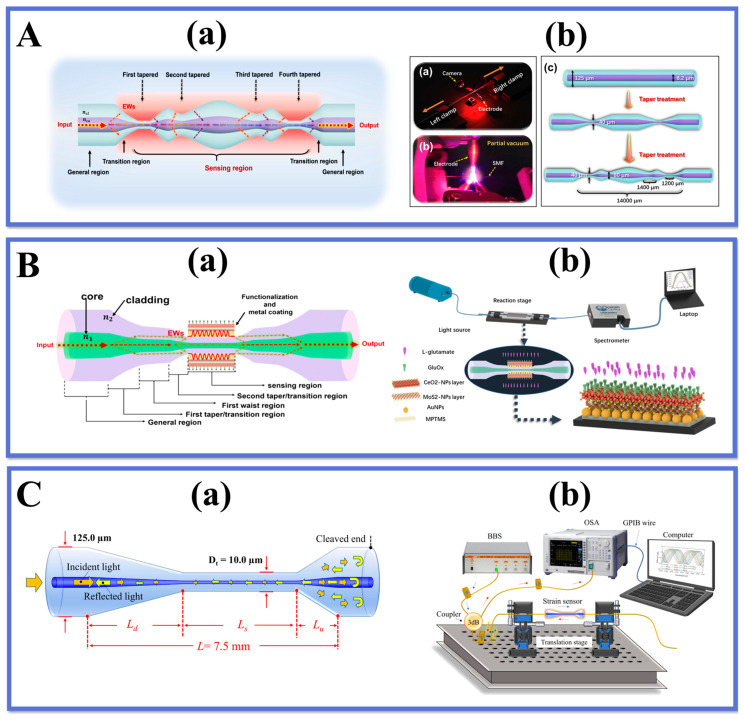
(**A**). Schematic of (**a**) the humanoid-shaped optical fiber sensor, (**b**) CMS machine tapering, and HTOF probe fabrication process. Reprinted with permission from Optics Express, Copyright 2023, Optica [149]; (**B**). schematic of (**a**) the tapered-in-taper fiber sensor, (**b**) experiment setup for ALT detection. Reprinted with permission from Measurement, Copyright 2023, Elsevier [150]; (**C**). schematic of (**a**) the asymmetric fiber sensor, (**b**) experiment setup for strain measurements. Reprinted with permission from Measurement, Copyright 2023, Elsevier [151].

Recent years have seen the emergence of innovative tapered structures in the field of fiber optic sensors. These structures offer new opportunities for expanding and developing applications for tapered fiber optic sensors. Figure 11 illustrates several typical examples of these new TOF structures, which can be categorized based on their different shapes. A novel simple, label-free human-shaped optical fiber structure was developed for the first time by Zhang et al. [149]. for detecting histamine concentration in food. the HTOF schematic and processing are shown in Figure 12A(a,b). By increasing the tapered processing, the structure’s tapered region is shaped to better excite the field in the sensing region and produce higher-power EWs. In addition, the structure has a larger surface area, which increases the contact area of the EWs with the environment. The results show that the sensor has good linearity for histamine in the range of 0–1000 µM with an LOD of 59.45 µM. In addition, the sensor has good reproducibility and repeatability, as well as immunity to other interfering substances. The sensor provides a novel and simple approach for developing fiber optic-based LSPR biosensors for food safety and biomedical applications.

Wang et al. [150] developed a novel LSPR-based sensor for the detection of alanine aminotransferase, a closely related clinical indicator of liver injury in human blood. The sensor employs a taper-in-taper (TIT) fiber optic structure with a structural schematic and experimental apparatus as shown in Figure 12B(a,b). This is the first time such a structure has been applied to biosensing. The structure was prepared by a three-electrode semi-vacuum taper pulling technique, and TIT was used to reprocess the single-taper structure to achieve a composite multi-taper structure. The variation of the transition region breaks the original TIR propagation pattern of the fiber, which facilitates the leakage of light inside the fiber. Increasing the complexity of the transition region further changes the propagation constant and excites additional higher-order modes to improve the sensitivity of the sensor, which is closely related to the unique structure of the transition region. The results show that the probe shows excellent linearity for the subsequently determined ALT concentration. In addition, the structure has high sensitivity and stability to determine its value for clinical application in the diagnosis of liver injury.

Mumtaz et al. [151] designed an ultrasensitive micro-strain sensor with an asymmetric fiber taper. The sensor was fabricated with a 10 μm ultra-thin asymmetric conical waist using SMF to form a Michelson interferometer. The schematic diagram of the modified structure and the experimental setup are shown in Figure 12C(a,b). The maximum strain sensitivity of the sensor is -39.77 pm/µε in the range of 0–1600 µε, and the long-term stability of the sensor for strain measurement is experimentally demonstrated. The sensor can realize the detection of small strains The sensor has the advantages of simple structure, easy fabrication, small size, and low cost, and can be widely used in the field of mechanical property testing at the micro and nano scale.

**Figure 13:**
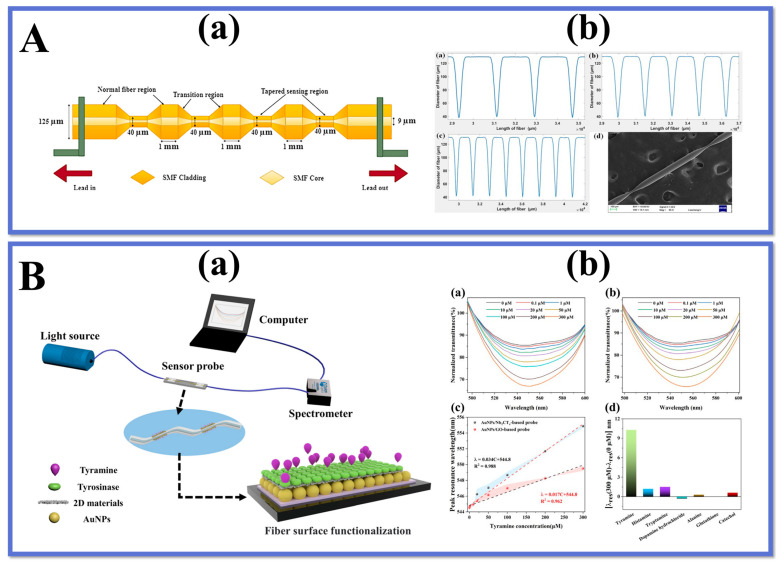
(**A**). Schematic of (**a**) the periodic fiber sensor, (**b**) diameter scanning results, and SEM image of periodic fiber. Reprinted with permission from Optics & Laser Technology, Copyright 2020, Elsevier [152]; (**B**). (**a**) experiment setup for tyramine test, (**b**) Test results for tyramine detection. Reprinted with permission from Applied Physics Letters, Copyright 2023, Elsevier [153].

Zhu et al. [152] introduced a tapered fiber optic sensor based on a periodic tapered structure for the detection of ascorbic acid. The probe structure and characterization diagrams are shown in Figure 13A(a,b). 

The innovation of this work is the fabrication of a periodic tapered structure on the fiber surface, which increases the interaction between the fiber and the external medium and improves the sensitivity of the sensor. By immobilizing AuNPs/GO and specific enzymes on the tapered structure, an effective biosensing platform is formed that can effectively adsorb and oxidize ascorbic acid. The quantitative detection of ascorbic acid concentration was achieved by measuring the change of fiber optic transmission spectra at different concentrations of the substance to be measured. It provides an effective strategy for the development of new high-performance fiber optic sensors and has potential applications in the field of rapid and accurate detection of ascorbic acid.

Li et al. [153] further optimized the tapered fiber interferometric structure using the cascade method and successfully fabricated a novel cascaded S-tapered fiber intermodal interferometric structure for detecting tyramine concentration. The dual S-tapered structure can provide more leakage EWs to enhance the light-matter interaction and to improve the sensor sensitivity. The two bending positions of the S-taper serve as two-beam coupling regions. At the first bending point, the light in the fiber core is coupled to the cladding, and the fundamental mode in the fiber core and the higher-order mode in the cladding are transmitted through the S-tapered fiber to produce a certain phase difference. In addition, as shown in Figure 13B(a), the sensor takes advantage of the excellent properties of Nb_2_CT_x_ MXene such as strong adsorption ability, large specific surface area, and excellent electrical conductivity, and modifies it with AuNPs and tyrosinase on the double S-tapered fiber to form an efficient biosensing interface. The results are shown in Figure 13B(b), with a sensor detection sensitivity of 34 pm/μM, a low detection limit of 0.35 μM, and a good selectivity. We also applied the sensor to real food samples to verify its reliability and practicality. The LSPR sensor with dual S-tapered optical fibers provides a novel and effective method to provide a new implementation for food safety monitoring.

Various shapes of TOFs have different advantages and disadvantages for fiber optic sensor applications. Therefore, when designing and developing different types of TOF structures, the requirements of the applications and the characteristics of different types of tapered fibers need to be considered. In addition, with the emergence of new structures of tapered fiber optic sensors, fiber optic sensor technology will be used in more fields. Furthermore, in combination with other sensor technologies, tapered fiber optic sensors can play an even greater role. Consequently, the future research direction not only lies in developing new structures and sensing applications, but also needs to explore the combination and integration of multiple sensing technologies to achieve more efficient and accurate measurement and detection.

## 7. Future Prospects

With the increasing demand in some new fields, the miniaturization of fiber-optic sensors with high sensitivity has clearly become a trend for future development. TOF sensors need to be integrated and miniaturized to meet the demand for miniaturization, portability, and multifunctionality of sensors. In addition, the performance of TOF sensors can be improved further. For example, the sensitivity, selectivity, stability, and other properties can be further improved by optimizing the structural design of tapered optical fibers, improving the preparation process, and exploring new surface modification methods. In addition, new fiber materials and fiber structures can be explored to meet the needs of different applications and achieve better sensitivity and resolution.

**Figure 14:**
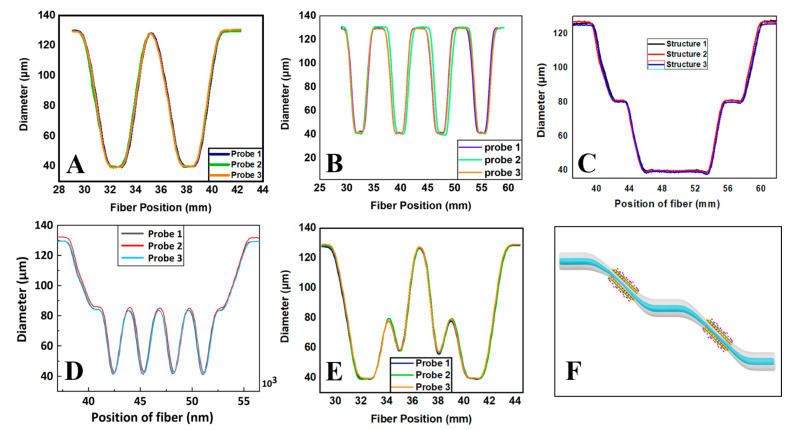
Graphs of diameter scan (**A**). humanoid-shaped; (**B**). taper-in-taper; (**C**). periodic taper; (**D**). dual-taper; (**E**). multi-taper-in-taper; and (**F**). schematic of double S-taper.

For further enhancement of the sensor performance, other innovative fiber optic structures have been investigated in depth, especially the fiber configuration shown in Figure 14, including dual-periodic tapered as well as quad-periodic tapered structure, taper-in-taper, and taper-in-multi-taper structure, humanoid-shaped and dual-periodic S-taper. Naturally, novel fiber structures are also being investigated, such as W-shapes and taper-in-taper with core mismatch structures, and it is reasonable to believe that these emerging fiber structures could play an important role in diverse applications.

On the other hand, although TOF sensors have a broad range of application prospects, it also faces some challenges. First, achieving large-scale preparation is still a challenge, and efficient and controllable preparation processes need to be sought to meet the needs of industrial production. Secondly, the stability of tapered fiber optic sensors during long-time use is also an important issue. Since the structure of tapered fiber optic sensors is relatively fragile and easily disturbed by the external environment, long-time use can lead to problems such as structural deformation or damage, thus affecting the performance of the sensor, which must be addressed further.

In conclusion, the TOF sensor is a promising sensor with a wide range of applications, but it still confronts hurdles in the preparation process, stability, challenging environmental applications, and performance improvement. These obstacles will be further solved and broken through in future research, with the continuous growth of science and technology, and it is believed that the bright development prospect of TOF sensing.

## 8. Conclusions

In this review, the progress of development, basic principles, and fabrication methods of tapered fiber optic-based sensors are discussed. It also focuses on the application of tapered fiber optic sensors in physical, chemical, gas, and biological fields, including research results in stress sensing, gas sensing, and biomolecule detection. These results thoroughly indicate that TOF sensors have evolved into a prospective novel sensors with a wide range of applications in various fields and promising future development prospects. On the other hand, some challenges for TOF sensors have been observed. Among them, the fabrication process and stability are the current issues that need to be overcome. To solve these challenges, more efficient and controllable fabrication processes are required to be developed to enhance the stability and durability of the sensors. 

The forthcoming innovative fiber-optic structures, including W-shape and taper-in-taper with core mismatch structures, will further explore the use of fiber-optic sensors for biosensing applications. In summary, TOF sensors have high application potential and development prospects, but still face some challenges. With the continuous development and innovation of technology, we believe that TOF sensors will be applied in more fields and become an important part of the new generation of sensors. It is believed that with continuous technical innovation and application demand, TOF sensors will be more widely used and innovated.

## Figures and Tables

**Table 1:** Applications of TOF-based sensors in physical parameter detection.

Measured Parameter	Fiber Structure	Sensing Principle	Linear Detection Range	Sensitivity	LOD	Ref.
Acoustic Vibration	Tapered-Tip Fiber	Radiated modes interference	1050 kHz	15.7 V/nm	0.1 nm	[100]
Humidity	Tapered dual side-hole fiber	Mach-Zehnder interferometer	30.3%–60.1% RH	−0.142 nm/% RH	n.r. ^a^	[101]
Liquid level	Single-mode taper-thin core taper single-mode fiber	Mach-Zehnder interferometer	0–15 mm	1.2416 nm/mm	n.r. ^a^	[102]
Load	Fiber tapered-loop probe	SPR	0–20 kPa	1.473 nm/kPa	n.r. ^a^	[103]
Magnetic Field	Tapered small core fiber	Modes interference	n.r.^a^	0.46 nm/mT	n.r. ^a^	[104]
RI/temperature	MMF-TSMF-MMF	Mach-Zehnder interferometer	1–1.001849 25–80 °C	−3244.22 nm/RIU −35.18 pm/°C	n.r. ^a^	[105]
RI	Tapered SMF	Mach-Zehnder interferometer	1.332–1.384 1.384–1.4204 1.4204–1.4408	415 nm/RIU 1103 nm/RIU 4234 nm/RIU	n.r. ^a^	[106]
RI	U-shape tapered plastic optical fiber	SPR	1.335–1.41	1534.53 nm/RIU	n.r. ^a^	[107]
Temperature	Taper-Like Etched Multicore Fiber	Mach-Zehnder interferometer	24–130 °C	89.19 pm/°C	n.r. ^a^	[108]
Temperature	Micro Taper In-Line Fiber	Mach-Zehnder interferometer	89–950 °C	0.113 nm/°C	n.r. ^a^	[109]

^a^ not reported.

**Table 2:** Applications of TOF-based sensors in chemical substances detection.

Measured Parameter	Fiber Structure	Sensing Principle	Linear Detection Range	Sensitivity	LOD	Ref.
Alcohol	Tapered fiber	SPR	Alcohol: 0–60% RI: 1.33–1.38	2350 nm/RIU	n.r. ^a^	[116]
Alcohol	Tapered MMF	Transmission intensity modulation	0–500 ppm	22 counts/ppm	n.r. ^a^	[117]
Co^2+^	Micro-tapered long-period fiber	Grating sensing	1 ppb–10^7^ ppb	2.4 × 10^−3^ dB/ppb	n.r. ^a^	[110]
Cu^2+^	Tapered fiber	Mach-Zehnder interferometer	0–1000 µM	0.0091 nm/µM	2.20 µM	[118]
Cu^2+^	Taper in taper	Mode-mode interference	0–0.1 mM	78.03 nm/mM	n.r. ^a^	[34]
Fluoride	Tapered Fiber Probe	EWs absorption	2.08 × 10^−6^ 2.005 × 10^−4^ M	n.r. ^a^	n.r. ^a^	[119]
Fluoride-ion	Taper Michelson interferometric sensor	Interference wavelength shift	0.01–0.10 ppb	3341.23 pm/ppb	n.r. ^a^	[120]
Nitrate	Tapered fiber	Polyurethane selective detection	n.r. ^a^	5.94 × 10^−2^ μW/ppm	n.r. ^a^	[121]
Pb^2+^	Tapered fiber	Interference wavelength shift	0.1–10^5^ ppb	0.03714 nm/ppb	0.0206 ppb	[115]
Sodium ions Manganese ions	Micro-tapered long period fiber	Grating sensing	1–10^6^ ppb	Sodium ions: 2.2 × 10^−3^ dB/ppb Manganese ions: 3.2 × 10^−3^ dB/ppb	3.2 ppb	[122]

^a^ not reported.

**Table 3:** Applications of TOF-based sensors in gas detection.

Measured Parameter	Fiber Structure	Sensing Principle	Linear Detection Range	Sensitivity	LOD	Ref.
Ammonia	Etched-tapered Single Mode Optical Fiber	EWs absorption	n.r. ^a^	300 au/%	0.00142%	[127]
Ammonia	Taper cascade	EWs absorption	n.r. ^a^	0.015 nm/ppm	n.r. ^a^	[128]
Ammonia	Tapered microfiber	EWs absorption	n.r. ^a^	1.30 pm/ppm	n.r. ^a^	[129]
Ammonia	Fiber fusion and taper	Transmission spectrum shift	0–10,476 ppm	0.58 pm/ppm	n.r. ^a^	[130]
Ammonia	Tapered fiber	Absorbance changes	n.r. ^a^	26.99 AU/%	13 ppm	[131]
Butane	Tapered Fiber	EWs absorption	n.r. ^a^	0.4812 a.u./vol.%	n.r. ^a^	[132]
Formaldehyde vapor	Tapered U-Shape Plastic Optical Fiber	EWs absorption	5–20%	0.00543 V/%	n.r. ^a^	[133]
Hydrogen gas	Tapered fiber	EWs absorption	0.125–2.00%	33.22/vol%	n.r. ^a^	[134]
Hydrogen gas	Tapered fiber	EWs absorption	0.125–2.00%	18,645%	n.r. ^a^	[123]

^a^ not reported.

**Table 4:** Applications of TOF-based sensors in biomolecule detection.

Measured Parameter	Fiber Structure	Sensing Principle	Linear Detection Range	Sensitivity	LOD	Ref.
Acetylcholine	Tapered/etched multicore fiber	LSPR	0–1000 μM	0.062 nm/μM	n.r. ^a^	[142]
Creatinine	CTC structure	LSPR	0–2000 µM	3.1 pm/µM	86.12 µM	[138]
Glucose	Tapered Optical Fiber	Evanescent field variations	n.r. ^a^	8.7 × 10^−3^ μW/mM	0.31 μW	[143]
Glucose	Tapered fiber	LSPR	n.r. ^a^	(5.01 ± 0.72) × 10^−3^ /a.u.(%)	n.r. ^a^	[144]
Hemoglobin	Micro-tapered long-period fiber grating	Optical waves interference	n.r. ^a^	3.14 mg/mL	0.057 mg/mL	[145]
p-Cresol	Tapered-in-Tapered fiber	LSPR	0–1 mM	3.8 pm/mM	0.14 mM	[146]
Cancer Biomarkers	Reflector-Less Shallow-Tapered Optical Fiber	Optical fiber dispersion	100 fM–10 nM	1.33 nm/RIU	16.4 pM	[51]
Triacylglycerides	Tapered Optical	EWs absorption	0–50 nM	0.9 nm/nM	0.23 nM	[147]
Protein	Tapered fiber	Interferometric effect	1–10 pM	1.02 nm/pM	n.r. ^a^	[148]

^a^ not report.

## Data Availability

Not applicable.
